# *ZmSOC1*, an MADS-Box Transcription Factor from *Zea mays*, Promotes Flowering in *Arabidopsis*

**DOI:** 10.3390/ijms151119987

**Published:** 2014-11-03

**Authors:** Suzhou Zhao, Yanzhong Luo, Zhanlu Zhang, Miaoyun Xu, Weibu Wang, Yangmin Zhao, Lan Zhang, Yunliu Fan, Lei Wang

**Affiliations:** 1 Biotechnology Research Institute/The National Key Facility for Crop Gene Resources and Genetic Improvement, Chinese Academy of Agricultural Sciences, Beijing 100081, China; E-Mails: zhao.404@163.com (S.Z.); yanzhongluo@163.com (Y.L.); xumiaoyun@caas.cn (M.X.); zhanglan01@caas.cn (L.Z.); fan_yunliu@163.com (Y.F.); 2 School of Life Science and Engineering, Southwest University of Science and Technology, Mianyang 621010, China; 3 Shenzhen Nongke Group CO., LTD, Shenzhen 518040, China; E-Mails: holyzzl@126.com (Z.Z.); wangwb628@sohu.com (W.W.); yangmin198715@126.com (Y.Z.)

**Keywords:** *Zea mays*, *ZmSOC1*, flowering, *Arabidopsis*, transcription factor

## Abstract

*Zea mays* is an economically important crop, but its molecular mechanism of flowering remains largely uncharacterized. The gene, *SUPPRESSOR OF OVEREXPRESSION OF CONSTANS 1* (*SOC1*), integrates multiple flowering signals to regulate floral transition in *Arabidopsis*. In this study, *ZmSOC1* was isolated from *Zea mays*. Sequence alignment and phylogenetic analysis demonstrated that the *ZmSOC1* protein contained a highly conserved MADS domain and a typical *SOC1* motif. *ZmSOC1* protein was localized in the nucleus in protoplasts and showed no transcriptional activation activity in yeast cells. *ZmSOC1* was highly expressed in maize reproductive organs, including filaments, ear and endosperm, but expression was very low in embryos; on the other hand, the abiotic stresses could repress *ZmSOC1* expression. Overexpression of *ZmSOC1* resulted in early flowering in *Arabidopsis* through increasing the expression of *AtLFY* and *AtAP1*. Overall, these results suggest that *ZmSOC1* is a flowering promoter in *Arabidopsis*.

## 1. Introduction

Flowering is regulated by both environmental and endogenous factors [1]. In *Arabidopsis thaliana*, six genetically defined pathways that control flowering have been identified: vernalization, photoperiod, ambient temperature, gibberellin, autonomous and age pathways [2]. Various signals in these pathways regulate flowering, but eventually converge to several integration factors, including *FLOWERING LOCUS C* (*FLC*), *FLOWERING LOCUS T* (*FT*), *SUPPRESSOR OF OVEREXPRESSION OF CO1* (*SOC1*) and *LEAFY* (*LFY*) [3,4,5,6,7].

The MADS-box family of transcription factors in plants is known for its role in developmental processes [8]. Most of the members of this family are involved in flowering regulation, vegetative development, meristem identity regulation, floral organ development and the formation of fruit and seed [9,10,11,12,13]. The MADS-box family contains a DNA binding domain of about 58 amino acids that binds DNA sequences known as the CArG box (CC(A/T)_6_GG) [14,15].

Models for the integration of genetic networks for flowering have been proposed in model plants, and *SOC1*, *FT* and *LFY* act as flowering pathway integrators [16,17,18]. *SOC1* encodes a MADS-box protein and contains a highly conserved DNA-binding domain. Studies have shown that *SOC1* integrates the GA pathway [19]. Furthermore, CO acts as a major positive factor underlying long day conditions, whereas the GA pathway acts as a positive factor underlying short day conditions [20,21,22]. *SOC1* interacts with multiple MADS box proteins, including *AGAMOUS-LIKE24* (*AGL24*), *FRUITFUL* (*FUL*) and *AP1*, and regulates the expression of several flowering genes, such as *SVP*, *AGL15*, *AGL18* and *SEP3*, by binding directly to regulatory sequences [20,23,24,25,26]. *SOC1* had been isolated from *Arabidopsis* [7,19,25], *Oryza sativa* [27,28,29], *Petunia hybrida* [9], *Citrus sinensis* [30], *Trillium camtschatcense* [31] and *Triticum aestivum* L. [32] and promoted flowering in transgenic *Arabidopsis*.

Maize is an economically important crop, and a comprehensive understanding of flowering behaviors is required to increase production and improve seed quality. However, the molecular mechanisms of flowering control in maize remain poorly understood. The *ZmMADS1/ZMM5*, which is a *SOC1*/*TM3*-like gene in *Zea mays*, is expressed in vegetative organs at a low level, increased in reproductive tissues and parallels the expression pattern in the development of *OsSOC1* and *AtSOC1* [7,28,33,34,35,36]. *ZmMADS1/ZMM5* not only contains the typical Mikc domain, but also has a highly conserved *SOC1* motif at the *C*-terminal, which uniquely exists in the *SOC1*/*TM3*-like gene [36,37]. Based on the similarity between *ZmMADS1/ZMM5* and *SOC1*, we renamed *ZmMADS1/ZMM5* as *ZmSOC1* [38].

In the present study, we isolated the *ZmSOC1* gene from *Zea mays* (B73). The protein sequence information, subcellular localization, transcriptional activation activity and expression pattern of *ZmSOC1* in maize were investigated. Our results show that *ZmSOC1* promotes flowering in *Arabidopsis* and suggest that the gene may play a similar role in maize.

## 2. Results and Discussion

### 2.1. Isolation and Characterization of ZmSOC1

**Figure 1:**
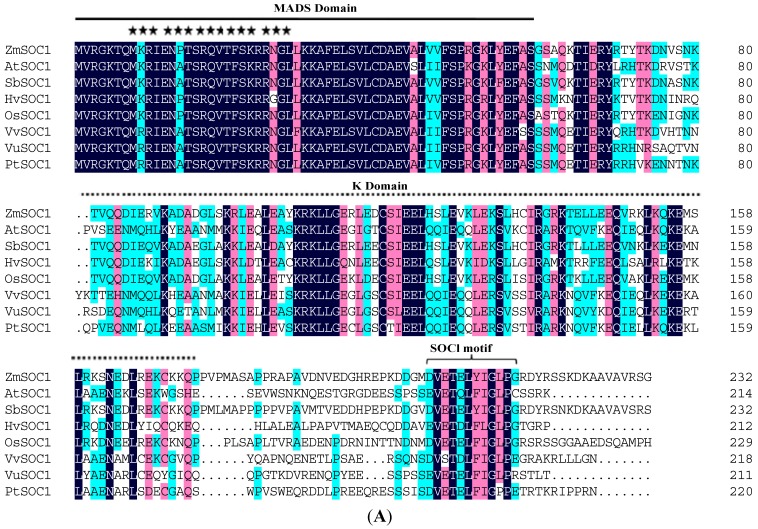

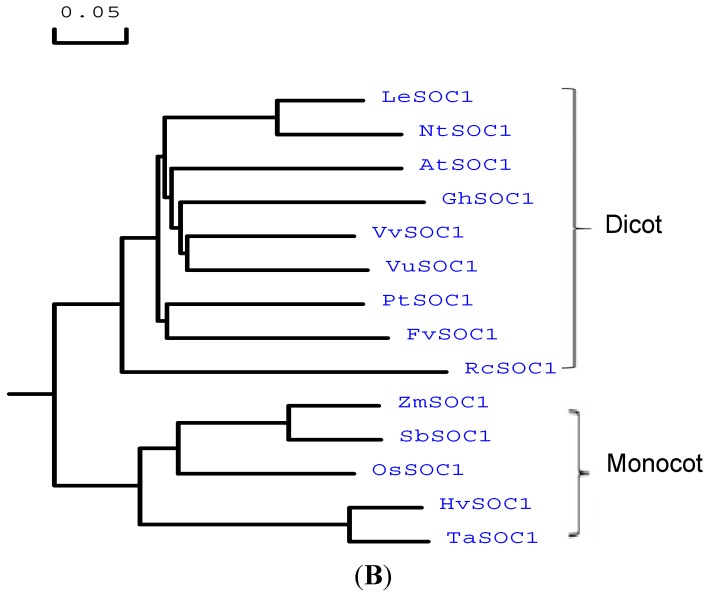
Bioinformatical analysis of *ZmSOC1.* (**A**) Amino acid sequence alignment of *ZmSOC1* and its homologs from other plant species. The proteins were initially aligned using DNAMAN software. Identical and similar amino acids are shown in black and red shading, respectively. The MADS domain, K domain and *SOC1* motif are marked with a solid line, dotted line and brackets, respectively. The locations of potential nuclear localization signals are marked with asterisks within the *ZmSOC1* amino acid sequence; (**B**) Phylogenetic analysis of *ZmSOC1* protein and its homologs from various plant species. The DNAMAN software was used for phylogenetic analysis. *ZmSOC1* protein could be divided into dicot and monocot clades. The accession numbers of the *SOC1*-like genes are as follows: *Arabidopsis thaliana SOC1*: *AtSOC1* (*NP_182090.1*); *Hordeum vulgare SOC1*: *HvSOC1* (*BAJ99551.1*); *Lycopersicon esculentum SOC1*: *LeSOC1* (*NP_001276829.1*); *Nicotiana tabacum SOC1*: *NtSOC1* (*CAA53782.1*); *Oryza sativa SOC1*: *OsSOC1* (*NP_001048801.1*); *Populus tremuloides SOC1*: *PtSOC1* (*AAP46287.1*); *Ricinus communis SOC1*: *RcSOC1* (*XP_002510866.1*); *Sorghum bicolor SOC1*: *SbSOC1* (*XP_002465961.1*); *Vitis vinifera SOC1*: *VvSOC1* (*NP_001267909.1*); *Fragaria vesca SOC1*: *FvSOC1* (*NP_001266966.1*); *Vigna unguiculata SOC1*: *VuSOC1* (*BAJ22387.1*); *Gossypium hirsutum SOC1*: *GhSOC1* (*AEA29618.1*); *and Triticum aestivum SOC1*: *TaSOC1* (*ABF57922.1*).

The ORF of the *ZmSOC1* gene was 696 base pairs (bp) in length and identical to *ZmMADS1* (GenBank ID: NM_001111682.1), encoding a protein of 232 amino acids with an estimated molecular mass of 26.4 kDa. A BLAST search of GenBank revealed that the *ZmSOC1* protein was similar to *SbSOC1* (87.50% identity), *OsSOC1* (75.11% identity), *AtSOC1* (53.99% identity), *HvSOC1* (65.09% identity), *VvSOC1* (54.17% identity), *PtSOC1* (52.05% identity) and *VuSOC1* (50.48% identity), respectively (Figure 1A). Multiple sequence alignment indicated that *ZmSOC1* contained a well-conserved MADS domain and a less-conserved K domain (Figure 1A). However, there are significant variations appearing in their *C*-terminals. In addition, a nuclear localization signal was predicted (Figure 1A). To investigate the relationship between *ZmSOC1* and other *SOC1*-like genes, a phylogenetic tree was constructed based on the amino acid sequences in their MADS-box domain (Figure 1B). The phylogenetic tree showed that all members could be divided into dicot and monocot clades, and the *ZmSOC1* protein belonged to the monocot clade together with other *SOC1* homologs from the *Gramineae* (Figure 1B).

### 2.2. Expression Profiles of ZmSOC1 in Maize

**Figure 2:**
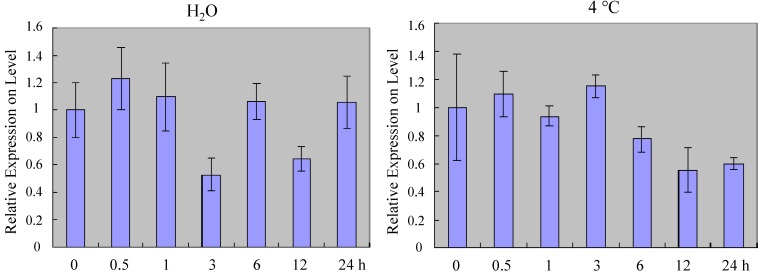

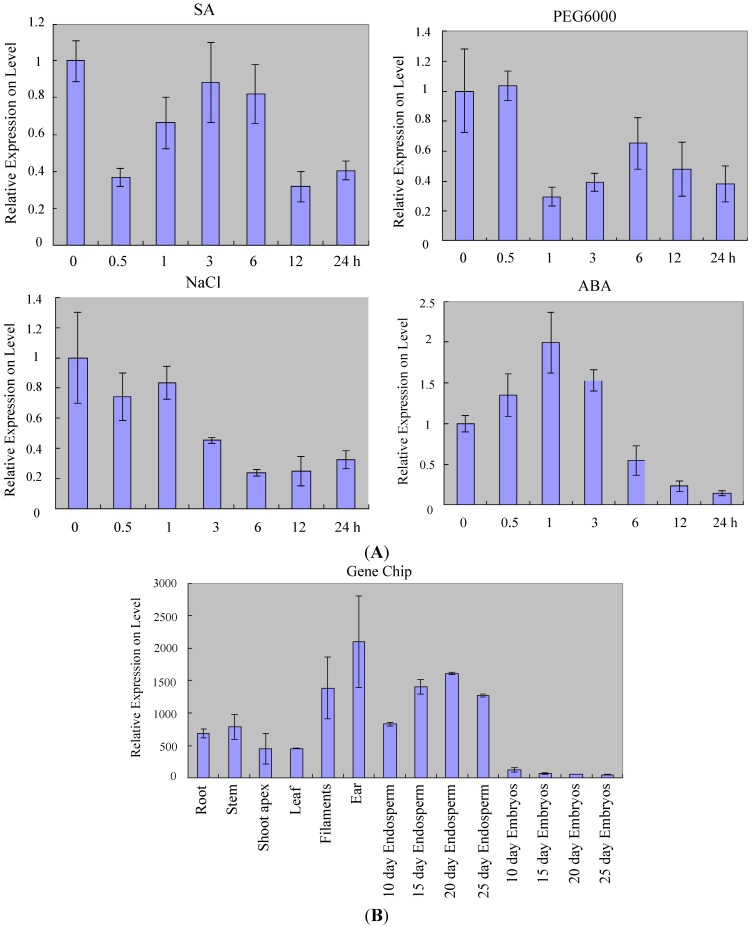
Expression profiles of *ZmSOC1.* (**A**) *ZmSOC1* expression under stress conditions. *ZmSOC1* expression was analyzed using qRT-PCR in Z31 leaves under different stress treatments (*i.e.*, NaCl (250 mM), PEG6000 (20%), Abscisic Acid (ABA, 100 µM), Salicylic acid (SA, 100 µM), 4 °C, and H_2_O without nutrients) for different period of time (0, 0.5, 1, 3, 6, 12 and 24 h); (**B**) *ZmSOC1* expression levels in maize tissues. The standardized data from the GeneChip of maize were used for the expression analysis; the data of eight tissues were extracted from root, stem, leaf, shoot apex, filament, ear, embryo and the endosperm compartment.

To explore whether *ZmSOC1* expression was regulated by abiotic stresses, maize seedlings were treated with NaCl, PEG6000, Abscisic Acid (ABA), Salicylic acid (SA), low temperature (4 °C) and water without nutrients. At 4 °C, *ZmSOC1* was slightly downregulated after 6 h; for NaCl treatment, *ZmSOC1* was significantly repressed after 3 h; for PEG6000 treatment, *ZmSOC1* was repressed quickly; for ABA treatment, *ZmSOC1* increased within 3 h and subsequently decreased after 6 h; for SA treatment, *ZmSOC1* expression decreased (but not significantly before 6 h), with a 40% reduction after 12 h, but the expression level of *ZmSOC1* was lightly changed in the water without nutrients (Figure 2A). Taken together, these results indicate that *ZmSOC1* expression was downregulated by the abiotic stresses.

To profile expression of *ZmSOC1* in different plant tissues and different developmental stages, we analyzed its expression using a GeneChip. *ZmSOC1* maintained expression at a moderate level with relatively stable expression in roots, stems, leaves and the shoot apex (Figure 2B). The expression level increased two- to three-fold in the filaments and ears, demonstrating that *ZmSOC1* plays an important role in maize floral organ development. Interestingly, its expression remained high for 10–25 days in endosperms, but in the embryos, the expression levels decreased to almost undetectable levels, indicating that *ZmSOC1* may be an essential factor for endosperm development (Figure 2B).

MADS-box gene-specific effects are closely related to internal and external environmental factors. Zhang and Forde [39] found NO_3_^−^ as a signaling molecule stimulating lateral root elongation in *Arabidopsis* and is dependent of the expression of the MADS-box gene, *ANR1*. Wang [40] found that the *RM1* (MADS-box) gene in rice callus tissue regulates plant cell dedifferentiation and re-differentiation processes and acts on target genes. After the target genes are activated, the cells transform into a distinct morphology. Overall, *ZmSOC1* as a MADS-box gene may play an important role in plant growth and developmental processes.

### 2.3. Subcellular Localization and Yeast Transcriptional Activation of ZmSOC1

**Figure 3:**
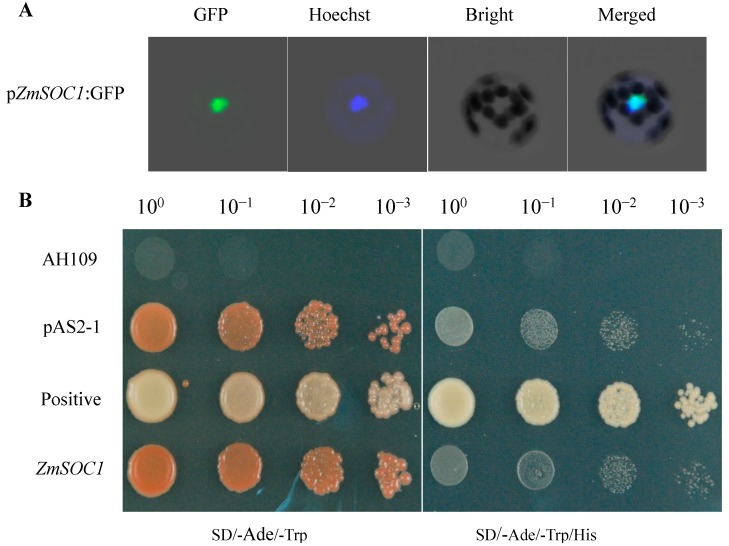
Subcellular localization and yeast transcriptional activation analysis of *ZmSOC1.* (**A**) Subcellular localization of the *ZmSOC1*:*GFP* fusion protein. The *Arabidopsis* protoplasts were transformed with the plasmid *ZmSOC1*:*GFP*. After the transfected protoplasts were incubated for 12–16 h at 22 °C in darkness, they were stained with 5 mg/mL Hoechst 33342 (CalBiochem) for 0.5–2.0 h to visualize the locations of the nucleus; (**B**) Analysis of *ZmSOC1* transcriptional activation activity in yeast. The empty plasmids, pAS2-1, pAS2-1:*ZmSOC1* and pGBK-GAL4-SV40-T53 (positive), were transferred into yeast strain AH109. Yeast were diluted in four gradient solutions.

*ZmSOC1* amino acid sequence analysis revealed a nuclear localization signal in the MADS-box domain (Figure 1A). To validate the subcellular localization of *ZmSOC1* protein, *ZmSOC1*:*GFP* was transformed into *Arabidopsis* mesophyll protoplasts. The fluorescence of *ZmSOC1*:*GFP* was localized in the nucleus as a blue spot according to Hoechst staining (Figure 3A). These data demonstrate that *ZmSOC1* entered the nucleus as a transcription factor and was involved in transcriptional regulation.

The transcriptional activation activity of *ZmSOC1* protein was analyzed using a yeast two-hybrid system. The recombinant plasmid pAS2-1:*ZmSOC1*, together with the positive control pGBK-GAL4-SV40-T53 and the negative control pAS2-1 plasmids, respectively, were transformed into the yeast strain AH109 containing the reporter gene HIS. All transformants grew well on the SD (Synthetic Dropout)/-Ade/-Trp media (Figure 3B), whereas on SD/-Ade/-Trp/-His media, only the positive control pGBK-GAL4-SV40-T53 transformants survived. These results demonstrate that *ZmSOC1* could not activate the expression of *HIS* genes in yeast cells, suggesting that *ZmSOC1* protein may have no transcriptional activation activity in yeast.

Transcription factors containing sequence-specific DNA-binding motifs are key molecular switches for nuclear localization, DNA binding and dimerization [24,25,41]. In the present study, a predicted nuclear localization signal was present in the MADS domain of *ZmSOC1* (Figure 1). As expected, the *ZmSOC1* protein mainly localized to the *Arabidopsis* nucleus (Figure 3). However, MADS-box transcriptional activation has not been widely investigated. The *C*-terminus in some MADS-box proteins functions as a core transcriptional activation domain [37,42]. In the present study, we demonstrated that the *ZmSOC1* protein had no transcriptional activation activity in yeast cells (Figure 3), similar to other MADS-box proteins, such as AGAMOUS (AG), APETALA3 (AP3) and PISTILLATA (PI) [43]. Because the *C*-terminal domain is the most divergent region among the MADS-box proteins, it is well established that some MADS-box proteins have transcriptional activity, while others do not [43,44].

### 2.4. Overexpression of ZmSOC1 Promotes Flowering in Arabidopsis

**Figure 4:**
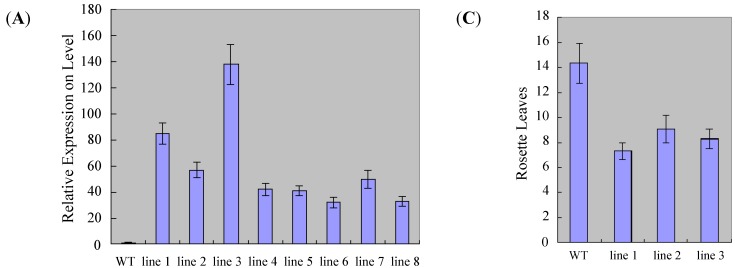

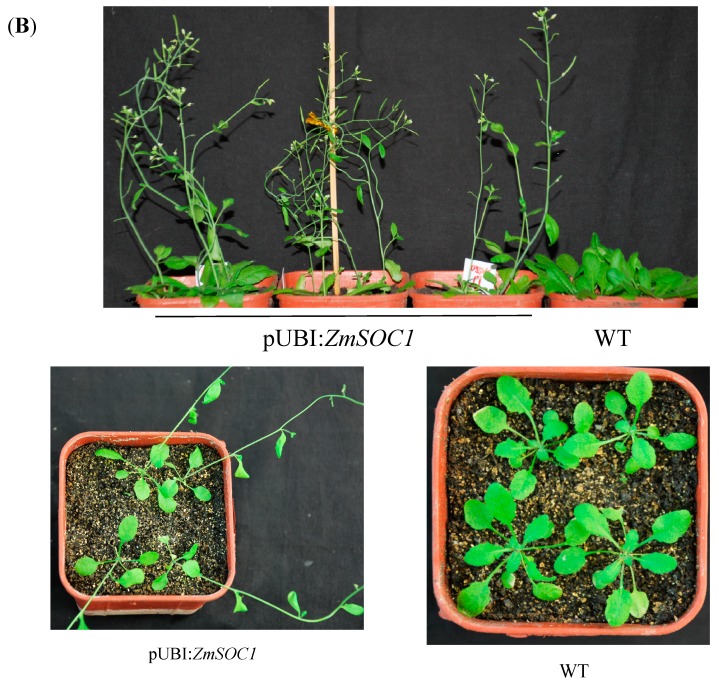
Overexpression of *ZmSOC1* promotes flowering in *Arabidopsis*. (**A**) *ZmSOC1* expression in transgenic plants. The expression level of *AtActin2* was used as an internal references. Values represent the means, and the error bars represent standard errors for three independent experiments; (**B**) The phenotype of *ZmSOC1* transgenic lines under the LD (Long-day) condition (14/10 h light/dark, 22 °C) after 30 days (**above**) and 40 days (**below**); (**C**) The number of rosette leaves during flowering in transgenic plants and wild-type plants.

To examine the role of *ZmSOC1* at flowering time, transgenic *ZmSOC1 Arabidopsis* plants driven by the Ubi promoter were generated. The transgenic plants were analyzed by qRT-PCR to confirm the expression level of *ZmSOC1*, and the transgenic lines all showed high *ZmSOC1* expression levels compared to wild-type (Figure 4A). Three independent T3 lines were selected for flowering time analysis under LD (Long-day) conditions. Compared to the wild-type, overexpression of *ZmSCOC1* significantly promoted flowering in *Arabidopsis*. The number of rosette leaves showing bolting ranged from 7.2 to 9.3 in *ZmSOC1*-overexpressing plants and was 14.5 for wild-type plants. In addition, the transgenic lines showed no morphological changes (excluding the leaves becoming slightly smaller). However, overexpression of another nine maize MADS-box genes was not observed to promote flowering in transgenic *Arabidopsis*. These results indicate that *ZmSOC1* functions as a flowering activator in *Arabidopsis*.

**Figure 5:**
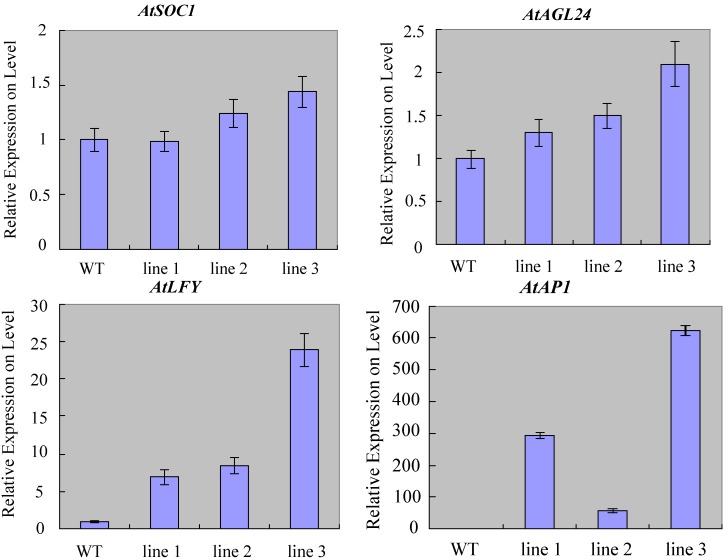
Expression patterns of *AtAGL24*, *AtSOC1*, *AtLFY* and *AtAP1* using qRT-PCR in wild-type and transgenic seedlings. The expression level of *AtActin2* was used as an internal reference. Values represent the means, and the error bars represent standard errors for three independent experiments.

To examine the molecular mechanisms by which *ZmSOC1* promotes flowering, the expression patterns of *AtAGL24*, *AtSOC1*, *AtLFY* and *AtAP1* were analyzed using qRT-PCR in wild-type and transgenic seedlings under LD conditions. *AtSOC1* expression levels in transgenic plants were similar to the wild-type. *AtAGL24* expression levels increased slightly, demonstrating that *ZmSOC1* overexpression did not influence the *SOC1* upstream gene in the flowering pathway. The downstream gene, *AtLFY*, was obviously upregulated in transgenic plants (Figure 5), suggesting that upregulation of *AtLFY* was the result of *ZmSOC1*. The expression of AP1 (downstream of *AtLFY*) was significantly upregulated in transgenic compared to wild-type plants (Figure 5). These results demonstrate that the high expression of *AtLFY* and *AtAP1*, which are upregulated by the overexpression of *ZmSOC1*, should be involved in the promotion of flowering time in transgenic plants.

The overexpression of *ZmSOC1* in *Arabidopsis* suggests that *ZmSOC1* plays an evolutionarily conserved role in the promotion of flowering. First, it caused early flowering (Figure 4), in agreement with previous studies [45,46,47]. Second, it upregulated the expression of *AtLFY* and *AtAP1* (Figure 5), which were directly or indirectly upregulated by *SOC1* during the floral transition [7,25,34]. Taken together, these results demonstrate that *ZmSOC1* may be a flowering promoter in maize. Because heterodimerization of *AtSOC1* and *AtAGL24* is a key mechanism activating *AtLFY* expression, we predicted that the interaction between *ZmSOC1* and *AtAGL24* would result in early flowering in transgenic *Arabidopsis* by activating *AtLFY* expression [7,25,34,48,49].

## 3. Experimental Section

### 3.1. Plant Material and Growth Conditions

*Arabidopsis thaliana* Columbia plants were grown in a greenhouse with a 14/10 h light/dark cycle at 22 °C. Seeds were sterilized for 10 min in 75% ethanol with 0.1% Triton X-100, for 5 min in 95% ethanol and finally washed with absolute ethanol on filter paper for drying. Seeds were germinated on half-strength Murashige and Skoog selective plates with 50 mg/L kanamycin. After 10 days of incubation in a growth chamber (14/10 h light/dark, 22 °C), resistant plants were transferred to soil.

### 3.2. Gene Cloning and Vector Construction

To clone the *ZmSOC1* gene, the *AtSOC1* protein (GenBank ID: NP_182090.1) was used for a BLAST search in the Maize Genetics and Genomics Database [50], and a protein (GRMZM2G171365) with high similarity to *AtSOC1* was identified. Total RNA was isolated using TRIzol reagent (Invitrogen, Carlsbad, CA, USA) from the ear of maize (B73). Total RNA was treated with DNase I (Promega, Madison, WI, USA), and first-strand cDNA synthesis was performed using a First Strand cDNA Synthesis Kit following the manufacturer’s instructions (Promega, Madison, WI, USA).

The *ZmSOC1* CDS was cloned into the pGWCVP64B vector using the Gateway recombination method, which contains an Ubi promoter and Nos terminator at the *C*-terminus. Based on the *ZmMADS1* sequence, the Building Gateway Enter Cloning System vector primers used were 5'-GGGGACAAGTTTGTACAAAAAAGCAGGCTTCATGGTGCGGGGCAAGACGCA-3' (forward) and 5'-GGGGACCACTTTGTACAAGAAAGCTGGGTTGCCTGACCTGACCGCCACTG-3' (reverse). The BP reaction mixture included attB-PCR product (100 ng), pDONR vector (100 ng) and BP Clonase™ Enzyme Mix (1 µL) (Invitrogen, Carlsbad, CA, USA). The LR reaction mixture included the Enter Cloning System Vector (100 ng), pGWVP64 vector (100 ng) and LR Clonase™ Enzyme Mix (1 µL) (Invitrogen, Carlsbad, CA, USA).

### 3.3. RNA Preparation and Gene Expression Assays

Total RNA was isolated from 3-week-old *Arabidopsis* and treated with DNase I. Quantitative real-time PCR (qRT-PCR) analysis was performed with an Applied Biosystems [51] Prism 7500 instrument and GoTaq^®^ qPCR Master Mix (Promega, Madison, WI, USA). For each genotype, three independent replicates consisting of five individual plants were used for analysis. The relative expression levels of target genes were calculated with the formula 2^−ΔΔ*C*t^ [52]. In *Arabidopsis*, we used qRT-PCR to investigate the genes expression levels, and the *AtActin2* gene was used as an internal control. The gene-specific primers used were 5'-CCCTCGTCGTCTTCTCCC-3' (forward) and 5'-CCATCCGCATCAGCTTTT-3' (reverse) for *ZmSOC1*; 5'-CCAACAGAGAGAAGATGACT-3' (forward) and 5'-ATGTCTCTTACAATTTCCCG-3' (reverse) for AtActin2; 5'-TCGAGTCAGCACCAAACCG-3' (forward) and 5'-TTGAGCATGTTCCTATGCCTTC-3' (reverse) for *AtSOC1*; 5'-ACTAGAGACGTTGGAAAGGG-3' (forward) and 5'-TCATTCCCAAGATGGAAGCCC-3' (reverse) for AtAGL24; 5'-AAGCCTAAAATGCGACACTACG-3' (forward) and 5'-GAGGATGAGCGTTAAAGACGG-3' (reverse) for *AtLFY*; and 5'-GGGATCAGCATAACCAAGGC-3' (forward) and 5'-ACGGGTTCAAGAGTCAGTTCG-3' (reverse) for *AtAP1*.

### 3.4. Bioinformatics Analysis

Genes homologous to *ZmSOC1* were identified using BLAST searches against GenBank [53], and potential sequence motifs were identified using SMART [54]. Protein sequence similarity ratio analysis was performed using DNAMAN software. Based on the e-value and identity, the orthologous of *ZmSOC1* were selected for the construction of the phylogenetic tree using DNAMAN software.

### 3.5. Subcellular Localization of ZmSOC1

The open reading frame (ORF) of *ZmSOC1* was amplified with the primer pair 5'-ATCTAGAATGGTGCGGGGCAAGACGCA-3' (containing a *Xba*I site) and 5'-ACCATGGTGCCTGACCTGACCGCCACTG-3' (containing a *Nco*I site). The PCR product was cloned into the pCAMBIA1302 vector *N*-terminus to generate *pZmSOC1:GFP*. *pZmSOC1:GFP* was transiently transformed into *Arabidopsis* mesophyll cell protoplasts using 10 μg plasmid DNA. For protoplast transient expression assays, mesophyll protoplasts were isolated from 6 to 8 4-week-old Arabidopsis rosette leaves, and the leaves were cut into 0.5–1 mm thin strips, then dropped into enzymatic solution (1.5% Cellulase R10, 0.4% macerozyme R10, 20 mM MES, 0.4 M mannitol, 10 mM CaCl2, 20 mM KCl, 0.1% bovine serum albumin, pH 5.7) and digested for 3 h. Plasmid DNA was prepared using the EndoFree Plasmid Midi Kit (CWBIO, Beijing, China). Protoplast transformation steps were performed as described by Yoo and Luan [55,56]. We washed protoplasts with W5 solution (2 mM MES, 154 mM NaCl, 125 mM CaCl_2_, 5 mM KCl, pH 5.7) and centrifuged them for collection. After cold treatment, protoplasts were resuspended with MMg solution (15 mM MgCl_2_, 4 mM MES, 0.4 M mannitol, pH 5.6), then an equal volume of PEG solution was added (40% PEG3350, 0.2 M mannitol, 0.1 M CaCl_2_), as well as 10 μg of the plasmid. After incubating for 10–15 min, transformed protoplasts were washed with W5, incubated with WI solution (0.5 M mannitol, 20 mM KCl, 4 mM MES, pH 5.7) at room temperature for 12–16 h in the dark. Hoechst 33342 dye (CalBiochem, San Diego, CA, USA) was added for 30 min, and the nuclei of living cells were stained blue [56]. *GFP* and Hoechst 33342 fluorescence in the nucleus was detected using LSM700 (ZEISS, Oberkochen, Germany).

### 3.6. Yeast Transcriptional Activation Assay

The full-length coding sequence of *ZmSOC1* was amplified with the primer pair 5'-ACCATGGTGCGGGGCAAGACGCAG-3' (*Nde*I site) and 5'-AGGATCCGCCTGACCTGACCGCCACT-3' (*BamH*I site). The PCR product was cloned into the pAS2-1 vector. For interaction studies, the plasmid was transformed into yeast strain AH109. Yeast cells were made competent for transformation by incubation in lithium acetate solution. Then transformation was performed by incubating the cells with transforming DNA, carrier DNA and PEG3350. The Yeast Transformation Kit (SIGMA, St. Louis, MO, USA) used the lithium acetate method. The transformed strains were cultured on SD/-Ade/-Trp and SD/-Ade/-His/-Trp synthetic complete drop-out media (SC drop-out) with 3-amino-1,2,4-triazole at 30 °C.

### 3.7. Stress Treatments in Maize

The maize inbred line Z31 was used in this study. Z31 seeds were washed with distilled water and set in a sprout machine at 28 °C for 4 days in the dark. Seeds with 2–3 cm germs were transferred to vermiculite to grow at 28 °C/22 °C in the 14/10 h light/dark cycle with nutrient solution irrigation. The nutrients in solution were (mmol·L^−1^): 0.75 K_2_SO_4_, 0.1 KCl, 0.25 KH_2_PO_4_, 0.65 MgSO_4_·7H_2_O and 0.2 EDTA-Fe; and (in µmol·L^−1^) 1.0 MnSO_4_·H_2_O, 1.0 ZnSO_4_·7H_2_O, 0.1 CuSO_4_·5H_2_O and 0.005 (NH_4_)_6_Mo_7_O_24_·4H_2_O [57]. After 10 days, the maize contained two leaves and a core stop providing nutrient solution. Subsequently, maize seedlings were treated with NaCl (250 mM), PEG6000 (20%), ABA (100 μM), and SA (100 μM) at 4 °C, with H_2_O as a control, and sampled at 0, 0.5, 1, 3, 6, 12 and 24 h. The samples were immediately frozen in liquid nitrogen, with three seedlings for each replicate.

### 3.8. Material Collecting for Microarray

Maize plants of inbred line B73 have been sequenced and have a mature GeneChip, so this was used to harvest these tissues. We harvested vegetative tissues at the big trumpet stage, including the roots, stems, leaves and the shoot apex, as well as reproductive tissues, including endosperms and embryos at 10, 15, 20 and 25 days after pollination; filaments and ear were harvested before pollination [58]. The above material was divided in three to get total RNA, and we used the Affymetrix GeneChip of maize to analysis in triplicate.

### 3.9. Statistics of Transgenic Arabidopsis in Phenotypes and Expression

The T3 transgenic and wild-type *Arabidopsis* were directly planted in nutritive soil, and total RNA was extracted from leaves after four weeks. qRT-PCR was used to analyze *ZmSOC1* expression levels in transgenic *Arabidopsis*. The number of rosette leaves was counted when these *Arabidopsis* began bolting, and 30 transgenic plants and wild-type *Arabidopsis* were counted, respectively.

## 4. Conclusions

*ZmSOC1* was isolated from maize and characterized. It showed high identity to other *SOC1* homologs and contained the well-conserved MADS domain and *SOC1* motif. The protein was localized to the *Arabidopsis* nucleus and showed no transcriptional activation activity in yeast cells. Overexpression of *ZmSOC1* promoted flowering through increasing the expression of *AtLFY* and *AtAP1* in transgenic *Arabidopsis. ZmSOC1* has a high expression level in floral organs, suggesting that *ZmSOC1* should be a flowering promoter in maize. These findings increase our understanding of the mechanism of flowering control in maize.
